# A Signature of Three microRNAs Is a Potential Diagnostic Biomarker for Glioblastoma

**DOI:** 10.52547/ibj.3671

**Published:** 2022-08-07

**Authors:** Ali Hosein Yazdi, Vajiheh Zarrinpour, Elham Moslemi, Mohamad Mahdi Forghani Fard

**Affiliations:** 1 Department of Biology, Damghan Branch, Islamic Azad University, Damghan, Iran;; 2 Cellular and Molecular Biology Department, Islamic Azad University, Tehran East Branch, Tehran, Iran

**Keywords:** Inflammation, Gastric cancer, Gene expression, Pattern recognition receptors, RNA-seq

## Abstract

**Background:**

Glioblastoma is the most common primary malignant neoplasm of the central nervous system. Despite progress in diagnosis and treatment, glioblastoma still has a poor prognosis. This study aimed to examine whether a signature of three candidate miRNAs (i.e. hsa-let-7c-5p, hsa-miR-206-5p, and hsa-miR-1909-5p) can be used as a diagnostic biomarker for distinguishing glioblastoma from healthy brain tissues.

**Methods:**

In this study, 50 FFPE glioblastoma tissue samples and 50 healthy tissue samples adjacent to tumor were included. The expression of each candidate miRNA (i.e. hsa-let-7c-5p, hsa-miR-206-5p, and hsa-miR-1909-5p) was measured using RT-qPCR. To show the roles of each miRNA and their biological effects on glioblastoma development and clinicopathological characteristics, *in silico *tools were used. ROC curves were performed to assess the diagnostic accuracy of each miRNA.

**Results:**

Based on the results, hsa-let-7c-5p and hsa-miR-206-5p were downregulated, while hsa-miR-1909-5p was upregulated in glioblastoma tumors compared to healthy samples. No association was detected between the expression of each candidate miRNA and sex. Except for hsa-let-7c-5p, other miRNAs did not correlate with age status. ROC curve analysis indicated that the signature of candidate miRNAs is a potential biomarker distinguishing between glioblastoma and healthy samples. Only hsa-miR-206-5p suggested the association with poor prognosis in glioblastoma patients.

**Conclusion:**

Our findings revealed that the signature of three miRNAs is capable of distinguishing glioblastoma tumor and healthy tissues. These results are beneficial for the clinical management of glioblastoma patients.

## INTRODUCTION

Glioblastoma, as the most frequent primary brain tumor and the most aggressive subtype of glioma, is characterized by a low survival rate (less than 12 months)^[1,2]^. MRI is commonly used for the initial diagnosis of glioblastoma, but in some cases, including low-grade glioma, it fails to make an accurate diagnosis^[4,5]^. Moreover, for further prognostic information, it is necessary to conduct histo-pathological and molecular analysis, which are mostly invasive^[3]^. Confounding factors, such as seudoprogression or pseudoresponse, are a major problems in the determination of brain tumor status^[6]^. Doing multiple biopsies also require invasive intracranial neurosurgery^[7]^ and are impossible in glioblastoma. Due to the intratumoral heterogeneity, glioblastoma biopsies are prone to sampling error^[8]^. To address these limitations, using biomarkers is recommended. 

Biomarkers can *ipso facto* help to more accurately diagnose and monitor glioblastoma patients at different stages of tumorigenesis^[9]^. Among various biomarkers, non-coding RNAs, particularly microRNAs (miRNAs or miRs), have attracted much attention^[10-14]^. MiRNAs are a small noncoding RNA of 18–25 nt that functions as a gene expression regulator at the post-translational level^[15,16]^. In other words, miRNAs function by binding to their target mRNAs and by suppressing the protein expression^[10,16]^. Aberrant expression or dysregulation of miRNAs can cause malignancy in glioma cells and may gives rise to glioblastoma development^[18]^. These molecules are the potential biomarkers for different cancers^[12,13]^ and play critical roles in cancer biology by controlling signaling pathways that contribute to tumor growth, angiogenesis, and immune invasion^[10,17]^. They are also detectable in blood and plasma, making them an appropriate biomarker for tumor diagnosis. Therefore, tissue-extracted RNAs can be the indication of the cellular status of the derived tissues; therefore, they can be used for diagnostic purposes^[17]^.

In the present study, we aimed to examine whether a signature of three candidate miRNAs (i.e. hsa-let-7c-5p, hsa-miR-206-5p, and hsa-miR-1909-5p) can be used as a diagnostic biomarker for distinguishing glioblastoma from healthy brain tissues. To this end, by literature reviews and *in silico* tools, we selected the above-mentioned miRNAs that were actively involved in glioblastoma development or pathogenesis. We also assessed the possible relationships between the expression pattern of these miRNA and the patients' clinicopathological features. To evaluate which miRNA discriminates glioblastoma from healthy brain tissues, a ROC curve analysis was used. Besides, *in silico* analysis was conducted to show probable targets and the functions of each candidate miRNA.

## MATERIALS AND METHODS

Patients 

FFPE samples were acquired from glioblastoma patients who were admitted by Imam Khomeini Cancer Institute, Tehran, Iran. Fifty patients with a definite clinical and imaging data diagnosis of glioblastoma were included in the study and subjected to surgery to remove injured tissues. As a common clinical procedure, MRI data were obtained for each patient. An experienced neuroradiologist blinded to the clinical or miRNA outcomes performed volumetric tumor analysis. Expert neuropathologists also assessed samples histopathologically based on histological grade classification provided by the World Health Organization^[19]^. Following the collection of glioblastoma tissues using standard operating procedures, a total of 100 tumor and healthy samples were gathered from the patients and healthy individuals, respectively. All the patients were newly diagnosed with glioblastoma, but healthy controls (n = 50) were those who had no positive history of brain pathology or malignancy; glioblastoma patients were subjected to surgery for different reasons besides malignancy, e.g. mild traumatic head injury.

RNA isolation and quality assessment

FFPE blocks were cut and transferred to a sterile tube for subsequent RNA isolation; the total RNA was isolated using TRIzol reagents (Invitrogen, VIC, Australia). Before isolation, FFPE tissue samples were rinsed multiple times by xylene to solubilize and remove the paraffin. The concentration of RNA samples was determined using the NanoDrop 2000c (Thermo Fisher Scientific, CA, USA), and the integrity of the samples was affirmed using 2% gel electrophoresis. All the samples were treated with RNase-free DNase (Ambion, Austin, TX, USA) to remove any probable DNA contaminations.

Candidate miRNA for experimental validation

Candidate miRNAs were selected, based on the literature reviews and *in silico* data mining, from miRNAs that may have a contribution to glioblastoma development. The selection criteria included: (i) the candidate miRNAs that were dysregulated and detectable in human glioblastoma, (ii) each candidate miRNA that has been annotated straightforwardly in miRBase 22.1, and (iii) the candidate miRNAs that might have been contributed to different signaling pathways in glioblastoma development or pathogenesis. Considering these criteria, hsa-let-7c-5p, hsa-miR-206-5p, and hsa-miR-1909-5p were chosen. 

**cDNA synthesis and RT-qPCR analysis**

Poly(A)tailing and cDNA synthesis were performed using MiR‐Amp Kit (ParsGenome, Tehran, Iran). The expression of each candidate miRNA was evaluated by RT-qPCR, which was carried out on an ABI StepOne Sequence Detection System (Applied Biosystems, NSW, Australia) under conditions reported in previous studies^[11,20-22]^. Melt curve analysis was used to assess the specificity of PCR. U48 snRNA was also utilized to normalize the relative expression of each candidate miRNA. To calculate the fold changes, we employed the 2^−ΔΔCt^ method^[23]^. The primer sequences used were as follows: *U48*‐forward: 5́-TGACCCCAGGTAACT CTGAGTGTGT-3́ and *U48*‐reverse: 5́-GCGTCGA CTAGTACAACTCAAG-3́, miR-let-7c-5p, forward: 5́-GGTGAGGTAGGTTGT-3́ and reverse: 5́-GTGCA GGGTCCGAGGT-3́, hsa-miR-206-5p: forward: 5́-GAATGTAAGGAAGTGTGTG-3́ and reverse: 5́-GAACATGTCTGCGTATCTC-3́, hsa-miR-1909-5p: 5́-TTGTGAGTGCCGGTGCCT-3́, and reverse: 5́-GTGCAGGGTCCGAGGT-3́.

Functional enrichment analysis

 In this study, we performed pathway analysis to determine which genes are targeted by the candidate miRNAs. To this end, bioinformatics analyses were performed using the Gene Ontology biological process and KEGG pathway options of the DIANA-miRPath v3.0^[24]^. We also used miRTargetLink Human to comprehensively explore human miRNA-mRNA interactions^[25]^. Furthermore, the miRWalk3.0^[26] ^was employed to determine which genes target each candidate miRNA. We considered the miRNA-gene pairs that were in common in at least five databases. To explore the clinical importance of miRNA expression, we used the expression data of the GEO database^[27]^. For this purpose, different data and several patients/controls were included, and a comparison was performed between glioblastoma and healthy samples. The sources and platforms used were as follows: source ID: GSE39486 and platform: GPL15829 for hsa-let-7c-5p, source ID: GSE135189 and platform: GPL20906 for hsa-miR-206-5p, and source ID: SRP262521 for hsa-miR-1909-5p. In total, 54 glioblastoma and 111 control samples were analyzed meticulously. 

Identification of candidate prognostic biomarkers

Glioblastoma is notorious for its poor prognosis^[28]^; thus, finding miRNA-based glioblastoma biomarkers can help follow up patients effectively^[29]^. In this study, as following up the glioblastoma patients were impossible, we used Pan-Cancer Tool^[30]^ to evaluate the association between the expression of each candidate miRNA and glioblastoma prognosis. We also calculated hazard ratio, 95% CI, and log-rank *p* values for each miRNA.

Statistical analysis

SPSS v.26.0 (IBM Corp., Armonk, NY, USA) and GraphPad Prism v.8.0 (GraphPad Software, Inc., La Jolla, CA, USA) were employed for expression analyses. Student's *t*-test was also used, and all experiments were repeated three times. The ROC curves were applied to calculate the diagnostic value for each candidate miRNA, particularly by calculating the AUC with at least 95% CI. *p *values less than 0.05 were considered statistically significant.

## RESULTS

Tissue samples and clinicopathological characteristics

We obtained 50 tumor and 50 healthy tissue samples from glioblastoma patients and healthy individuals, respectively. An overview of the clinicopathological characteristics of the glioblastoma patients are represented in Supplementary Table 1. The mean patient age at the time of diagnosis was 59.38 ± 10.28 years ranging between 46 and 77 years (59.38 ± 10.28 years ranging between 46 and 77 years in male patients and 60.24±10.24 years ranging between 46 and 76 years in female patients). The mean overall survival time was 14.5 months ranging between 10 and 19 months (14 months between 10 and 18 months for male and 15 months between 11 and 19 months for female patients). No differences were found in the mean age and overall survival rate between the two groups of patients. The distribution of age and sex was matched between the male and female groups. 

Expression analysis of candidate miRNA 

**Fig. 1:**
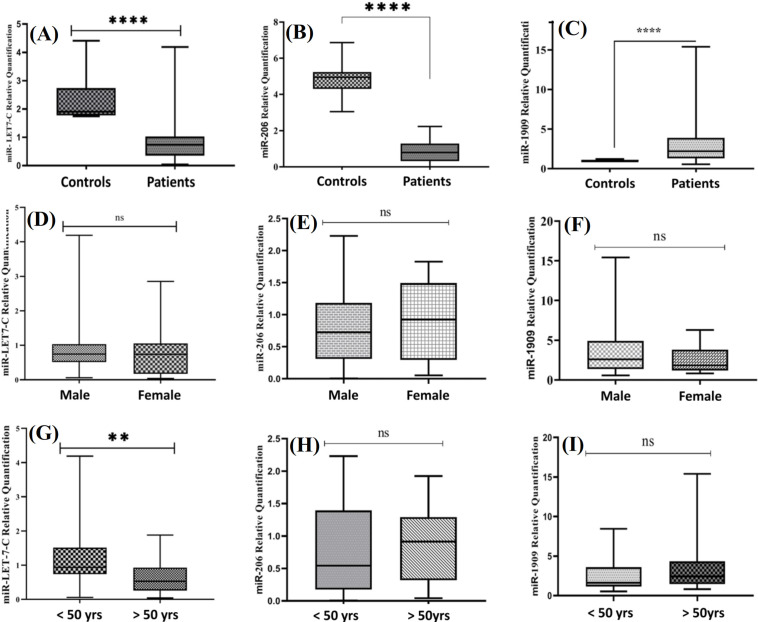
The expression analysis of the candidate miRNAs in glioblastoma and healthy tissues. (A) hsa-let-7c-5p is downregulated in glioblastoma patients compared to the control group, (B) hsa-miR-206-5p is downregulated in tumor samples, and (C) hsa-miR-1909-5p is upregulated in tumor tissues compared to the healthy tissues; (D and F) there are no associations between the expression of each candidate miRNA and the participants’ sex; (G) expression of hsa-let-7c-5p is correlated with the ‘age’ factor, i.e. higher expression of this miRNA was observed in patients who were <50 years; (H and I) no association was detected between the expression of hsa-miR-206-5p and -1909-5p and age factor. ^**^ *p* < 0.01; ^****^p < 0.0001; ns: not significant.

The expression of the selected candidate miRNAs was analyzed 50 tumor and 50 healthy tissue samples. RT‐qPCR was used to determine the expression levels of each candidate miRNA in the putative samples. To calculate the RT-qPCR efficiency, we prepared fivefold serial dilutions of cDNA samples and exploited standard curves. The efficiency was measured based on the slope of the standard curve and using the equation E = 10^(-1/slope)^. The amplification efficiency of each miRNA and internal control was equal with a high linear correlation, implying the validity of the assay. Furthermore, a dissociation curve was used to verify the uniqueness and specificity of amplified products. The single and sharp peaks of the melting curves demonstrated no primer‐dimer or nonspecific products. It has been indicated that the integrity of miRNAs derived from FFPE tissues has prone to modification and fragmentation in FFPE tissues^ [31]^; however, due to the small size of these molecules (about 19-22 nt), there was a little chance of degradation. Our results displayed that hsa-let-7c-5p and hsa-miR-206-5p were downregulated in glioblastoma samples, while hsa-miR-1909-5p was upregulated in tumor samples compared to healthy tissues (*p *< 0.000; Fig. 1A-1C). 

Correlation between miRNA expression and clinicopathological features

Association between the expression of each miRNA and clinicopathological characteristics (age, gender, tumor location, tumor size, and overall survival rate) of glioblastoma patients was statistically analyzed. Except for hsa-let-7c-5p, which was expressed abundantly in patients aged <50 years, expressions of hsa-miR-206-5p and hsa-miR-1909-5p did not show any association in terms of sex and age (Fig. 1D-1I). Based on the statistical analysis, no considerable association was also observed between hsa-miR-206-5p and hsa-miR-206-5p expressions with tumor location and overall survival rate (*p *> 0.05). However, a significant relationship was detected between hsa-let-7c-5p expression and tumor location, tumor size, and overall survival rate (*p *= 0.013, 0.037, and 0.014, respectively). 

miRNA biomarkers in glioblastoma

**Fig. 2:**
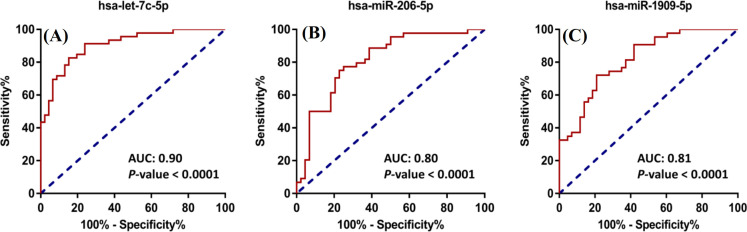
ROC curve analysis performed for each candidate miRNA. Vertical axes represent the sensitivity, while the horizontal ones show specificity. The AUC was used to measure the diagnostic performances for each candidate miRNA. We considered miRNA as a biomarker if its calculated AUC was above 0.5. The AUC scores calculated for hsa-let-7c-5p (A), -miR-206-5p (B), and -miR-1909-5p (C) were 0.90, 0.80, and 0.81 (*p* <0.0001), respectively.

The sensitivity and specificity of each candidate miRNA were analyzed by the AUC-ROC curve, which, in turn, showed that each miRNA potentially was a tumor and diagnostic biomarker for glioblastoma. We considered the miRNAs as the diagnostic biomarkers when their calculated AUC was above 0.50. The calculated AUC for hsa-let-7C-5p, hsa-miR-206-5p, and has-miR-1909-5p were 0.90, 0.80, and 0.81, respectively (*p *< 0.0001; 95% CI; Fig. 2A-2C).

Analysis of functional enrichment analysis

**Fig. 3:**
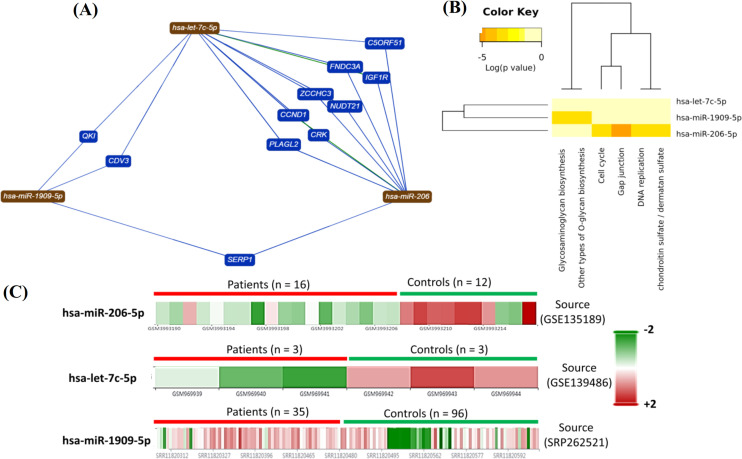
Functional enrichment analysis. (A) miRNA-mRNA interactions were obtained using miRTargetLink Human. All findings were confirmed using miRWalk 2.0; (B) a miRNA *vs.*GOSlim groups heatmap was drawn using DIANA-miRPath v3.0 that in turn shows the enrichment level in Gene Ontology categories of the candidate miRNAs; (C) unsupervised hierarchical clustering analysis confirmed that each miRNA is differentially expressed in glioblastoma compared to healthy tissues. The heatmaps were obtained using the GEO database.

**Fig. 4:**
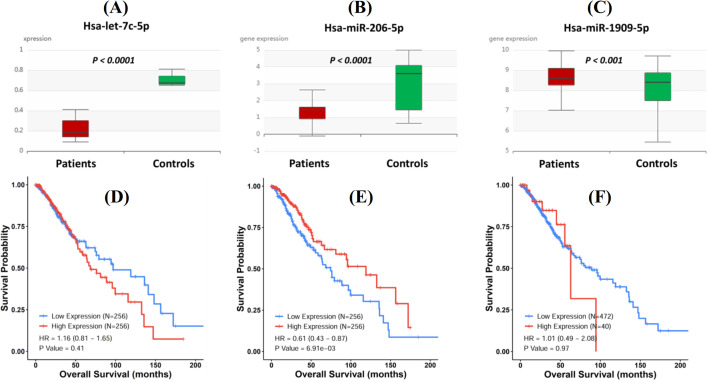
Expression and prognostic values of each miRNA calculated and confirmed using *in silico *tools. The expression of each candidate miRNA was determined using the GEO database. (A) hsa-let-7c-5p, (B) hsa-miR-206-5p, and (C) hsa-miR-1909-5p. The glioblastoma dataset from Kaplan Meier Plotter was used to test for the survival prediction capacity of each candidate miRNA. Samples were divided into two distinct groups, including low (blue) and high (red) expression groups for each target. In total, at least 500 individuals were analyzed. Hazard ratio and *p *value for each association are shown within each plot. (D) hsa-let-7c-5p; (E) hsa-miR-206-5p; and (F) hsa-miR-1909-5p. *p* < 0.05 was considered as valid data.

In this study, we used miRTargetLink Human to indicate the integrated network for the candidate miRNAs and their potential targets (Fig. 3A; Supplementary Fig. 1). The tool could identify several genes that were potential targets for hsa-let7-c-5p, hsa-miR-206-5p, and hsa-miR-1909-5p (Fig. 3A). By applying DIANA-miR Path v.3.0, we exhibited that the candidate miRNAs could contribute to different cell signaling procedures such as cell cycle, insulin-like growth factor signaling, cell growth, and proliferation (Fig. 3B). MiRWalk 2.0 was also employed to confirm the identified possible interactions between the candidate miRNAs and their targets. Hierarchical clustering analysis of candidate miRNA indicated that the miRNAs discriminate between glioblastoma tumors and healthy tissues (Fig. 3C). GEO database was implemented to evaluate the expression levels of each candidate miRNA in glioblastoma (Fig. 4A-4C). The identified and predicted functions of each miRNA were determined using the KEGG database (Supplementary Fig. 1).

Survival analysis of the miRNA-based signature

Overall glioblastoma survival was determined by *in silico* tools, which showed to be low in cases with the aberrant expression of each miRNA candidate. The follow up duration was determined at 200 months (Fig. 4D-4F). Our data showed that hsa-miR-206-5p might be a prognostic glioblastoma biomarker (hazard ratio = 0.61). Regarding other miRNAs, i.e. hsa-let-7c-5p and hsa-miR-1909-5p, the potentiality of being a prognostic biomarker was not significantly detected (Fig. 4D-4F).

## DISCUSSION

Despite advances in surgical and adjuvant treatments, glioblastoma patients still suffer from poor prognoses^[32]^. MiRNA-based biomarkers have indicated some advantages of being easily obtainable^[12]^ and existing throughout the disease course and tumor stage^[33]^. Recently, miRNAs have been identified as promising non-invasive biomarkers showing tumor development in the early stages and improving glioblastoma diagnosis and prognosis^[34]^. Herein, based on the literature reviews and data mining using *in silico* tools, we selected three miRNAs, including hsa-let-7c-5p, hsa-miR-206-5p, and hsa-miR-1909-5p, that may contribute to glioblastoma development. Different miRNAs have been identified to be able to successfully discriminate glioblastoma; thus, we conducted an investigation into whether a signature of these candidate miRNAs can be a new diagnostic biomarker. To fill this gap, the expression of each candidate miRNA was determined using RT-qPCR, and their associations with clinicopathological features were analyzed.

Hsa-let-7c is a vital tumor suppressor miRNA playing a proactive role in the inhibition of proliferation, migration, and invasion of lung cancer cells^[35]^; this miRNA is somehow linked to the etiology of recurrent lung cancer, although its exact mechanism is still unclear. Luo *et al.*^[36]^ have demonstrated that hsa-let-7c-5p may regulate *CyclinD1* expression via the Wnt/β-catenin signaling pathway in osteoblasts, suggesting that this miRNA likely has a function in cell cycling of tumor cells. Hsa-let-7c-5p is downregulated in recurrent glioblastoma than in primary glioblastoma samples^[37]^. Also, hsa-let-7c inhibits metastasis in colorectal cancer, and its downregulation stimulates the expression of K-RAS, MMP11, and PBX3 and promotes cell migration, glioblastoma, and invasion^[38,39]^. Therefore, hsa-let-7c-5p downregulation may contribute to glioblastoma recurrence via triggering glioblastoma cell migration and invasion. While many studies have highlighted different roles of miRNA in cellular and molecular events, its functions in glioblastoma are still obscure. In this study, we showed that this miRNA is downregulated in patients' samples than in the control tissues, which is consistent with the data derived from the GEO database (Fig. 4A-4C) and verifies the tumor suppressor roles of this miRNA. The AUC-ROC curve analysis proposed that this miRNA is a reliable biomarker with favorable sensitivity and specificity that can distinguish between glioblastoma and healthy tissues. However, the prognostic potentiality was not detected *in silico*. 

Hsa-miR-206 has been demonstrated to suppress cell proliferation in gastric cancer, at least partially by targeting the CyclinD2^[40]^. Similarly, the expression of this miRNA is downregulated in breast cancer^[41]^, laryngeal cancer^[42]^, rhabdomyosarcoma^[43]^, colorectal cancer^[44]^, and prostate cancer^[45]^. These findings showed a tumor-suppressive role for hsa-miR-206-5p, which activates apoptosis and inhibits tumor cell migration^[46]^.Overexpression of hsa-miR-206-5p gives rise to the decreased expression of metabolic genes, NADPH production, and ribose synthesis in mouse models^[47]^. Hao *et al.*^[48]^ indicated that the expression of hsa-miR-206 decreased considerably in glioblastoma tumor samples and pertinent cell models compared to the control tissues/cells. They also demonstrated that hsa-miR-206 is a tumor suppressor, and its inhibition provides a prerequisite for glioblastoma development and progression. Our findings were in line with these results. Using AUC-ROC curve analysis, we showed that hsa-miR-206-5p is a promising biomarker that can discriminate the glioblastoma from healthy tissues. Also, using *in silico* data, we displayed that this miRNA is a probable prognostic biomarker for glioblastoma; however, further studies are needed to reveal the exact molecular mechanisms whereby this miRNA functions in glioblastoma development/progression.

Hsa-miR-1909-5p is upregulated in pediatric dysembryoplastic neuroepithelial tumors, casting light on its oncogenic functions^[49]^. Braoudaki *et al*.^[49] ^have exhibited that this miRNA is a potential biomarker distinguishing between the tumor types and normal tissues. It has also been recognized that hsa-miR-1909-5p is significantly upregulated in less aggressive rectal cancer patients^[50]^. Hsa-miR-1909 is expressed in the early stages of gastric cancer^[51]^; however, regarding glioblastoma, there is scant information on the roles of this miRNA in the development or progression. Li *et al.*^[52]^ have elucidated that miR-1909-5p is significantly upregulated in a glioblastoma patient. It has been identified using a high-throughput technique that miR-1909-5p is upregulated in glioblastoma tumors^[53]^. Our results underscored that miR-1909-5p has oncogenic roles in glioblastoma development, though further studies are required to arrive at a conclusion and to explore the detailed underlying molecular mechanisms. Recently, it has been demonstrated that miR-1909-5p may play a tumor-suppressive role in the etiology of Parkinson's disease, i.e. its downregulation was identified in postmortem samples of patients with Parkinson's disease^[54]^. Overall, we demonstrated that hsa-miR-1909-5p is a diagnostic biomarker in glioblastoma and is upregulated in glioblastoma patients, as proved by *in silico *tools. Long-term follow ups did not show any prognostic biomarker potentiality for this miRNA. In this study, we also showed that hsa-let-7c-5p, hsa-miR-206-5p, and hsa-miR-1909-5p target different genes, most of which are involved in cell cycle, insulin-like growth factor signaling, cell proliferation/growth, and cell migration. This observation could substantiate that the signature of these miRNA may represent different cellular processes that are often dysregulated in glioblastoma.

This study has some limitations; for instance, obtaining direct samples from glioblastoma tumors needs surgery. It would be feasible and more convenient if we checked the expression of the signature in serum, plasma, or cerebrospinal fluids. Therefore, further studies seem to be imperative to determine the exosomal levels of this signature to show whether this can be used as a non-invasive method to discriminate between tumor and healthy samples or not. Likewise, as *in silico* analysis showed, the signature targets a substantial number of genes; hence, miRNA and gene expression profiling is necessary to determine which genes are targeted by the signature. Conducting larger investigations with more diverse samples would help confirm our findings.

The present study revealed that the signature of three miRNAs (i.e., hsa-let-7c-5p, -206-5p, and -1909-5p) is capable of distinguishing glioblastoma tumor and healthy tissues; however, further studies are essential to unveil the detailed molecular mechanisms whereby these miRNAs play roles in glioblastoma development.

## DECLARATIONS

### Ethical statement

The above-mentioned sampling protocols were approved by the Ethics Committee of Islamic Azad University, Damghan, Iran (ethical code: IR.IAU.DAMGHAN.REC.1399.018). All donors voluntarily gave their written informed consent before sampling. 

### Data availability

The raw data supporting the conclusions of this article are available from the authors upon reasonable request.

### Author contributions

AHY: data acquisition, supervision, formal analysis, writing review and editing; VZ: data acquisition, supervision, and writing original draft; EM: data acquisition, supervision, and writing original draft; MMFF: data acquisition. All authors have read and approved the final version of the manuscript.

### Conflict of interest

None declared.

### Funding/support

This study did not have a financial grant and was conducted by Mr. Hossein Yazdi to complete his Ph.D. dissertation.
